# Study on the detection of water status of tomato (*Solanum lycopersicum* L.) by multimodal deep learning

**DOI:** 10.3389/fpls.2023.1094142

**Published:** 2023-05-31

**Authors:** Zhiyu Zuo, Jindong Mu, Wenjie Li, Quan Bu, Hanping Mao, Xiaodong Zhang, Lvhua Han, Jiheng Ni

**Affiliations:** ^1^ School of Agricultural Engineering, Jiangsu University, Zhenjiang, China; ^2^ Key Laboratory of Modern Agricultural Equipment and Technology, Ministry of Education/High-tech Key Laboratory of Agricultural Equipment and Intelligence of Jiangsu Province, Jiangsu University, Zhenjiang, China

**Keywords:** multimodal deep learning, water status, tomato, depth images, nondestructive detection

## Abstract

Water plays a very important role in the growth of tomato (*Solanum lycopersicum* L.), and how to detect the water status of tomato is the key to precise irrigation. The objective of this study is to detect the water status of tomato by fusing RGB, NIR and depth image information through deep learning. Five irrigation levels were set to cultivate tomatoes in different water states, with irrigation amounts of 150%, 125%, 100%, 75%, and 50% of reference evapotranspiration calculated by a modified Penman-Monteith equation, respectively. The water status of tomatoes was divided into five categories: severely irrigated deficit, slightly irrigated deficit, moderately irrigated, slightly over-irrigated, and severely over-irrigated. RGB images, depth images and NIR images of the upper part of the tomato plant were taken as data sets. The data sets were used to train and test the tomato water status detection models built with single-mode and multimodal deep learning networks, respectively. In the single-mode deep learning network, two CNNs, VGG-16 and Resnet-50, were trained on a single RGB image, a depth image, or a NIR image for a total of six cases. In the multimodal deep learning network, two or more of the RGB images, depth images and NIR images were trained with VGG-16 or Resnet-50, respectively, for a total of 20 combinations. Results showed that the accuracy of tomato water status detection based on single-mode deep learning ranged from 88.97% to 93.09%, while the accuracy of tomato water status detection based on multimodal deep learning ranged from 93.09% to 99.18%. The multimodal deep learning significantly outperformed the single-modal deep learning. The tomato water status detection model built using a multimodal deep learning network with ResNet-50 for RGB images and VGG-16 for depth and NIR images was optimal. This study provides a novel method for non-destructive detection of water status of tomato and gives a reference for precise irrigation management.

## 1 Introduction

The global tomato (*Solanum lycopersicum* L.) harvest areas reached approximate 5.052 million hectares in 2020 (FAO, 2021). Irrigation management affects the growth and development of tomatoes (Ma et al., 2022; Zhao et al., 2022). Both excessive or deficient water supply have influence on the yield and quality of tomatoes. Deficient water supply may lead to water stress, and excessive water can affect root respiration (Liu et al., 2019) which results in the waste of water resources. Water status of tomatoes can provide guidance for irrigation management (Scalisi et al., 2019; Li et al., 2021). It has a key role in future irrigation management, therefore, research on water status detection in tomato is urgent.

At present, research on crop water status detection have received increasing attentions from scholars. The leaves of plants are sensitive to water change, and the drying method can measure the water content of leaves or the whole plant, which can obtain the accurate water content. Leaf water potential measured by the pressure chamber method, or the small liquid flow method is also an indicator to reflect the water status of plants. However, the pressure chamber method and the small liquid flow method involve taking samples from crops, which is not only time-consuming and labor-intensive, may causes some damage to the crops and cannot be applied to real-time irrigation. To avoid damage to the crops, many researchers have been dedicated to the real-time nondestructive detection of the water status of the crops. It mainly includes judgments based on RGB images (Li et al., 2020), terahertz spectra (Li et al., 2020), NIR hyperspectral (Duarte-Carvajalino et al., 2021), infrared thermography (Khorsandi et al., 2018), 3-D images (Zhao et al., 2012) and the variation of stem diameter (Meng et al., 2017). Currently, RGB images used for crop water status detection commonly apply deep learning networks to classify the collected RGB images for detection, and deep learning networks usually utilize CNNs. However, RGB images are easily affected by light and background (Hu et al., 2019). The waveband of terahertz spectroscopy has sensitive absorption of moisture, and researchers have studied the variation of terahertz parameters of crops with different water status and constructed a detection model of crop water status, which has high detection accuracy in the laboratory; however, terahertz spectroscopy cannot be environmentally controlled in actual detection as in the laboratory, and water in the environment can also interfere with the detection (Wu et al., 2022). Hyperspectral images are rich in information and can predict the moisture content of crops based on NIR hyperspectral images. Infrared thermography detects the temperature information of the crop and thus determines the water status of the crop. NIR spectroscopy and infrared thermography for crop moisture detection are based on the principle of thermal radiation, which is influenced by environmental changes (Zhang X. et al., 2021). It is possible to determine the water status of a crop based on its 3-D morphology, but it is difficult and complicated to obtain 3-D images and process them (Zhao et al., 2016). The change of stalk diameter is closely related to the crop water status, which is an effective indicator to detect the crop water status, but the stalk will harden when the crop grows and gradually stops changing, and the position of the measuring instrument needs to be changed regularly (Namba et al., 2018). Besides, the stem thickness measurement sensors are more expensive (Wakamori et al., 2020).

In recent years, great progress has been made in the field of artificial intelligence (Baltrusaitis et al., 2019). With the proposal of precision agriculture, artificial intelligence has been used in agriculture in many applications. In comparison with the traditional methods, the method using deep learning can get more accurate detection results(Garillos-Manliguez & Chiang, 2021). Multiple modal data can be obtained for the same object, and the data of different modalities can be complemented with each other to make the data more comprehensive and help improve the accuracy by fusing the data of different modalities (Garillos-Manliguez & Chiang, 2021).

The objective of this research is to detect the water status of tomatoes by fusing RGB, NIR and depth image information through deep learning. It will provide a novel method for non-destructive detection of water status of tomatoes and give a reference for irrigation management.

## 2 Materials and methods

### 2.1 Experimental design

**Figure 1:**
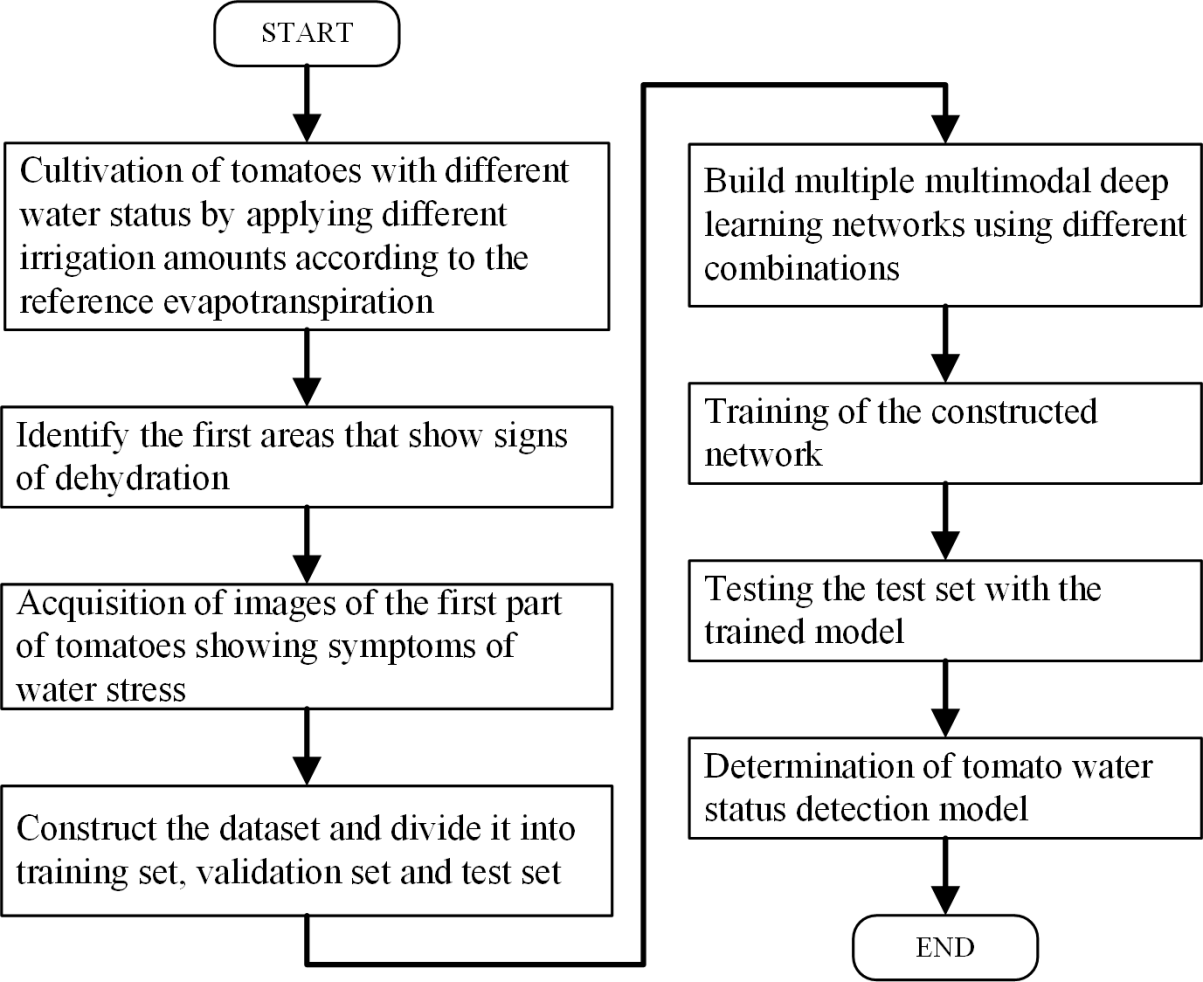
Flow chart of tomato water status detection model.

**Figure 2:**
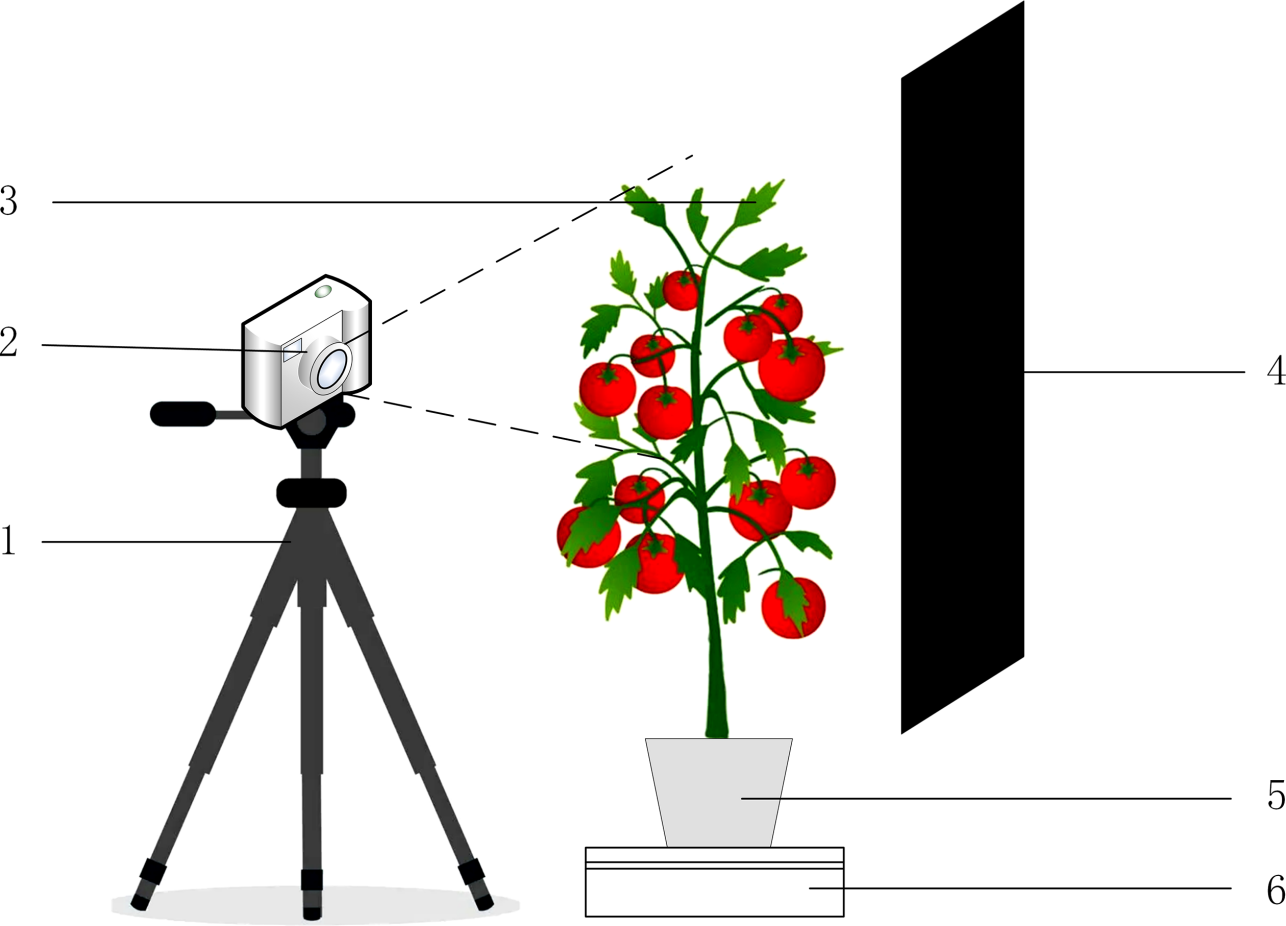
Schematic diagram of taking tomato images. (1) Camera tripod. (2) RealSense camera. (3) Tomato plant. (4) Black cardboard. (5) Flowerpot. (6) Motorized turntable.

The water status of the test samples was controlled according to the Penman-Monteith equation for irrigation at different percentages to cultivate tomatoes with different moisture contents. The data collection section introduced the instrumentation, collection methods and the processing of the data set. The tomato water status detection network construction section investigated the performance of different combinations of neural networks, and the idea of tomato water status detection model construction is shown in **Figure 1**:

1. To determine the parts of tomatoes that first exhibited water deficiency symptoms during water deficiency so that images of the appropriate parts can be acquired later. To cultivate tomatoes with different water status, RGB images, depth images and NIR images of the upper leaves of tomatoes were captured using a RealSense camera (it can capture RGB images, depth images and NIR images) as shown in **Figure 2**. The captured images were made into a dataset and divided into a training set, a validation set and a test set.
2. In order to obtain the most suitable detection model for the water status of tomatoes, the detection model constructed using one kind of image was first trained, then the detection model constructed using two kinds of images was trained, and finally the detection network constructed using three kinds of images was trained.
3. The three types of trained multiple detection models were tested on the test set, and each detection model was compared and analyzed to select the appropriate detection model.

### 2.2 Cultivation of the experimental samples

The experiments were conducted in the Venlo continuous glass greenhouse at Jiangsu University from June 2021 to January 2022, and the tomato variety used was “Pink Crown F1” (Shouhe). The substrate used was perlite and the nutrient solution formulation was “Yamazaki Nutrient Solution Formula” (Zhang X. et al., 2021). Five irrigation levels were set, with five tomato plants at each irrigation level, irrigated at 50%, 75%, 100%, 125%, and 150% of the reference evapotranspiration of tomatoes, corresponding to the water status of tomatoes as severely irrigated deficit, slightly irrigated deficit, moderately irrigated, slightly over-irrigated, and severely over-irrigated. The reference evapotranspiration of tomato was calculated by the modified Penman-Monteith equation. According to Xu et al. (2020), the reference evapotranspiration of tomato is defined as Eq.(1).


(1) $$ET_{r}=\frac{0.408\Delta (R_{n}-G)+\gamma \frac{1713(e_{a}-e_{d})}{T+273}}{\Delta +1.64\gamma }\times K_{c}$$

where *ET_r_* is the reference evapotranspiration (mm/d), Δ is the slope of saturated water vapor pressure versus temperature curve, *R_n_* is the net radiation (MJ/m^2^d), *G* is the soil heat flux (MJ/m^2^d), *γ* is the psychrometer constant (kPa/°C), *e_a_* is the average saturated water vapor pressure (kPa), *e_d_* is the actual water vapor pressure (kPa), *T* is the average daily air temperature (°C), and *K_c_* is the crop coefficient of tomato at different growth stages (0.75 at seedling stage, 1.05 at flowering stage and 0.8 at fruiting stage).

Every morning, about one hour after sunrise time, the amount of irrigation for the day was calculated according to Eq.(1) and then irrigated into the tomato cultivation flowerpot at once.

### 2.3 Tomato image acquisition and dataset production

#### 2.3.1 Instrumentation

The D435i RealSense camera (Intel, USA) is a viable tool for outdoor, close-range agricultural phenotyping tasks (Vit and Shani, 2018). The camera was therefore selected to capture RGB images, NIR images and depth images of the tomato canopy, with resolutions up to 1920×1080 for RGB images and 1280×720 for depth images, and a depth measurement range of 0.2m-10m, which can be modified within the range according to actual needs. To avoid the camera’s IR projector interfering with the NIR image, the IR projector is turned off before the NIR image is acquired.

The test platform was Dell Precision 7920 workstation with Intel Xeon 4110 processor, NVIDIA Quadro P4000 graphics card, 8GB of graphics memory, 64GB of computer memory, and Windows 10 Professional Workstation Edition operating system. The deep learning network was written in Python, Python version was 3.7. The deep learning framework was PyTorch, version 1.7.1 accelerated with CUDA 11.0 and cuDNN8.0.5.

#### 2.3.2 Determination of shooting position

To determine the shooting position of tomatoes, the position where tomatoes first showed water deficit symptoms were explored. Eight tomato plants were cultivated individually, four of which were irrigated normally and the other four were subjected to water stress treatment with suspension of irrigation, while all other managements were the same. After the start of the experimental treatment, images were taken every hour at three different positions, including the upper, middle and lower parts of the tomato plants.

**Figure 3:**
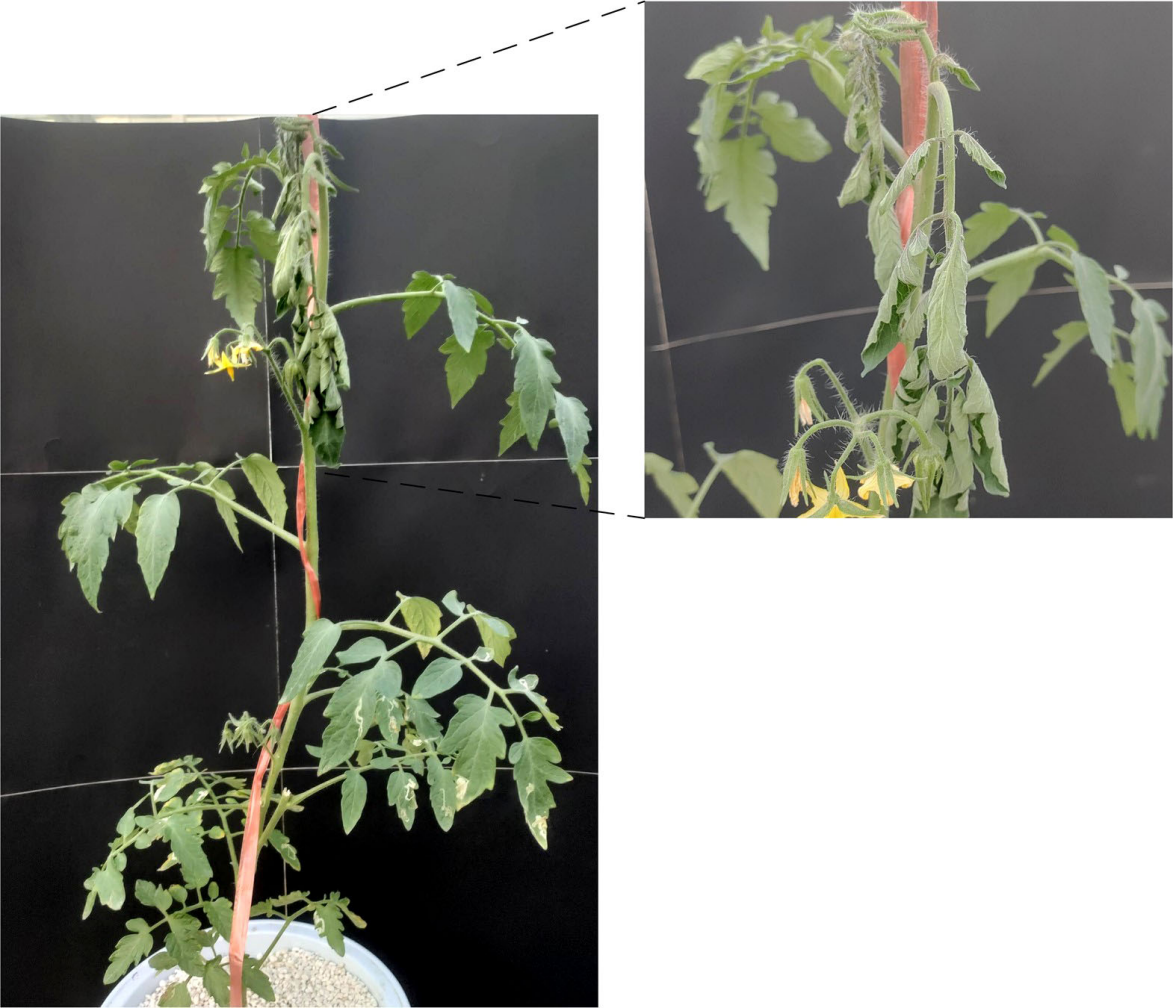
An image of a tomato plant treated by water stress.

**Figure 4:**
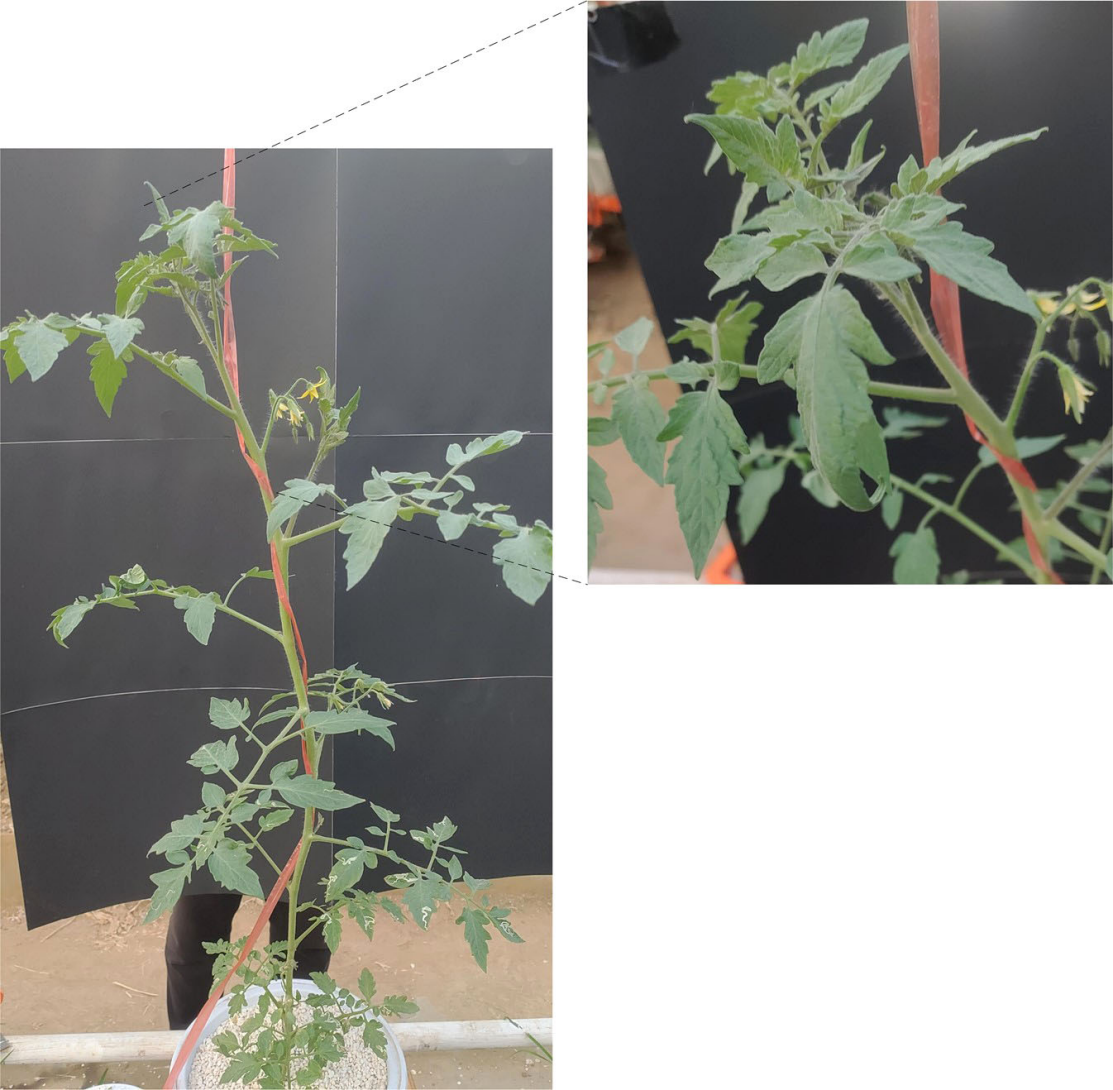
An image of a control tomato plant.

The upper leaves of the water stress treated tomato plants showed wilting first, while the middle and lower leaves were in better condition than the upper leaves, as shown in **Figure 3**. **Figure 4** shows an image of a control tomato plant for the same period, where no water stress symptoms were observed throughout the plant. When tomato was subjected to water stress, the upper leaves were the first to show water stress symptoms. The images of the upper leaves of tomato plants were selected to better detect the water status of the plants earlier. Therefore, it was determined that the upper part of the tomato plant was the target region for water status detection.

#### 2.3.3 Image acquisition and dataset creation

**Figure 5:**
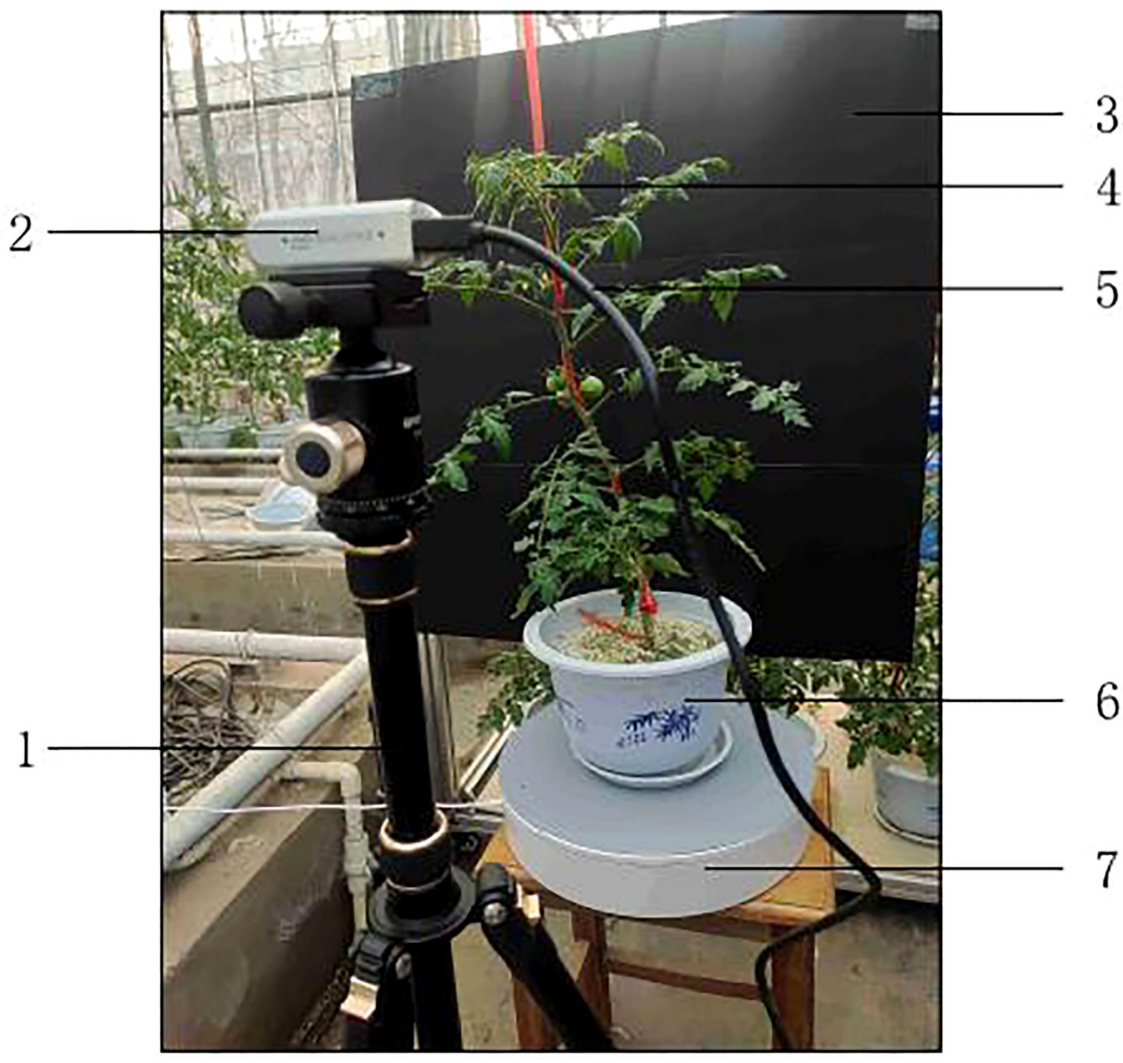
Experiment Scene of image acquisition. (1) Camera tripod. (2) RealSense camera. (3) Black cardboard. (4) Tomato plant. (5) Data cable. (6) Flowerpot. (7) Motorized turntable.

The image acquisition test scenario is shown in **Figure 5**. The RealSense camera was fixed by a camera tripod, the distance was about 30 cm from the foremost part of the tomato and aimed at the upper leaves of the tomato. The RealSense camera was connected to the computer via a data cable with a Type-C interface on the RealSense camera side and a USB 3.0 interface on the computer side. The tomatoes were placed on a motorized turntable, which was stopped for 3 seconds every 1/96th of a revolution, and photographed using the RealSense camera. This was done to obtain more images on the one hand and to ensure that images from different angles of the tomatoes were captured on the other hand. A piece of black cardboard was placed behind the tomato plant to reduce the interference of the background. Before training, 10% of the image edge was cut to avoid the edge exceeding the black background plate, adjust the size of the clipped RGB image and near-infrared image to 640×480, adjust the size of the depth image to 424×240, and remove image noise using Gaussian filter (Li et al., 2019).

**Figure 6:**
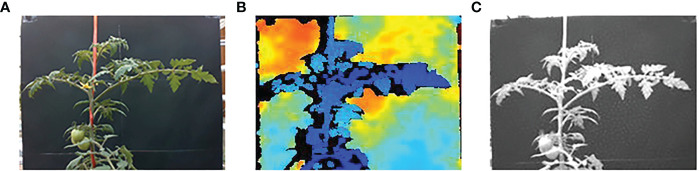
Acquired images. **(A)** RGB image. **(B)** Depth image. **(C)** NIR image.

Image acquisition was performed after 7 days of water treatment. A total of 21,600 sets of images were acquired as a data set for the experiment, and a set of images contained RGB images, depth images, and NIR images, and the images acquired for each moisture state were 4320 sets. As shown in **Figure 6**, (A) is the RGB image, (B) is the visualized depth image, and (C) is the NIR image. The training set accounted for 70% of the data set, the validation set accounted for 10% of the data set, and the test set accounted for 20% of the data set. The images in the training set and the test set are from different tomato plants.

### 2.4 Construction of the water status detection

Multimodal data fusion can be mainly divided into three main types: early fusion, late fusion and hybrid fusion (Bayoudh et al., 2021; Joshi et al., 2021; Zhang Y. et al., 2021). Early fusion involves the fusion of the features extracted from the data collected by the sensors and then the detection model is used for classification, which is also known as feature fusion. Late fusion refers to processing the data of each modality individually, training them independently, and then calculating the result according to the weight of each network calculation. It is also called decision layer fusion, which will ignore the relevant features between modalities and have a large information loss (Choi and Lee, 2019). Some studies have shown that early fusion is superior than late fusion. Hybrid fusion combines early fusion and late fusion, while different data have different dimensions and scales, making fusion more difficult.

**Figure 7:**
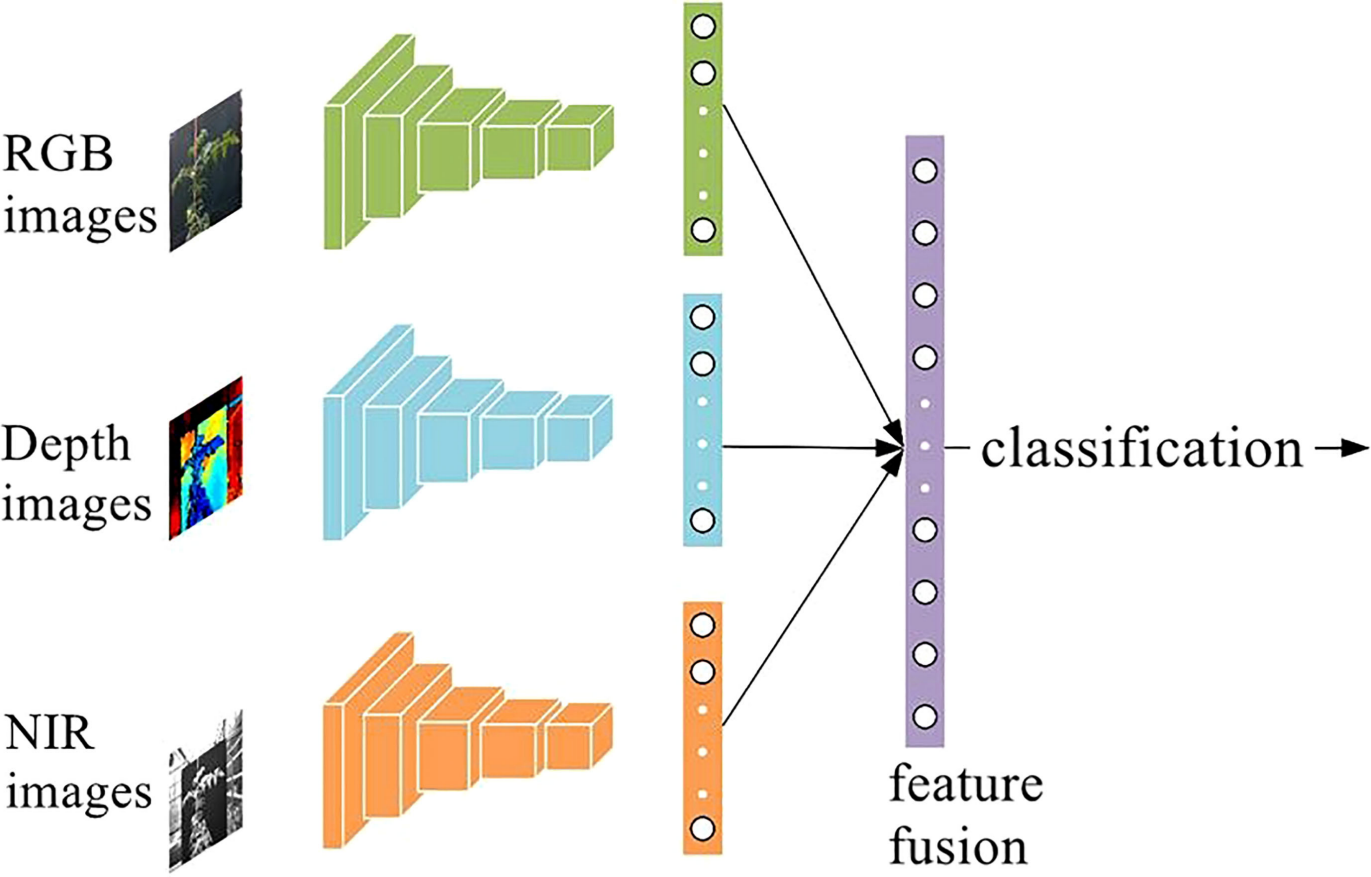
Diagram of the deep learning network structure of detection of crop water status.

In this study, early fusion was used and CNNs was applied to extract data features. The extracted image features were then fused and classified by a classifier to construct a deep learning network for tomato water status detection. Image features were extracted using VGG-16 and ResNet-50, and the main reasons for using these two CNNs were: VGG-16 and ResNet-50 had good performances in multiple datasets (Gao et al., 2019). It had been widely used in recent years and had also achieved great performance. To perform feature fusion, the fully connected layers of VGG-16 and ResNet-50 were used for fusion, VGG-16 and ResNet-50 with the fully connected layer with the output of detection results removed were used. the features in the fully connected layer were rich in semantic features, and these semantic features had a significant role in image classification (Gao et al., 2019). The feature size shape extracted by VGG-16 was 1×1×4096 and the feature size extracted by ResNet-50 was 1×1×2048. The extracted features were stitched together using data that had gone through the pooling layer. The constructed deep learning network was trained and the optimal combination was selected according to the detection effect. The structure of the constructed deep learning network for water status detection is shown in **Figure 7**.

## 3 Results

### 3.1 Experimental evaluation indicators

To be able to evaluate the detection performance of each combination network and then select the optimal combination, recognition accuracy was used as an evaluation index in this study. Accuracy recognition is the most intuitive way to understand the performance of the detection network and is an extremely important evaluation index, which can be calculated by Eq.(2).


(2) $$Accuracy=\frac{P_{c}}{P_{ALL}}\times 100\%$$

where *P_c_* is the number of correctly classified and *P_ALL_* is the number of total samples.

### 3.2 Single-modal deep learning network

**Figure 8:**
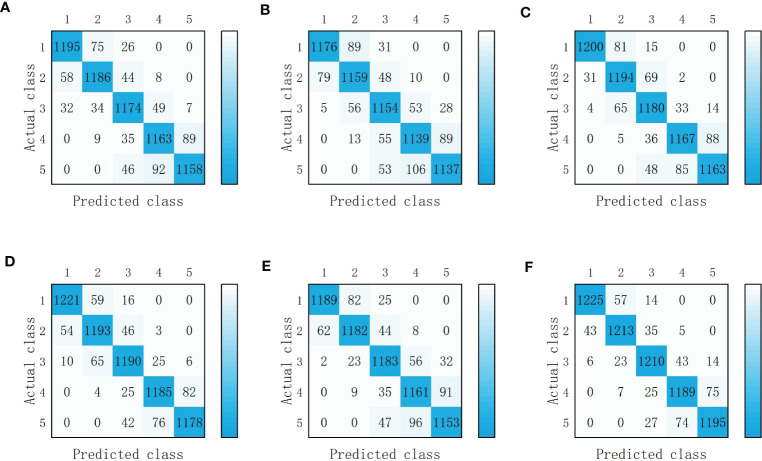
Detection results of single-modal deep learning model. **(A)** VGG-16(RGB images). **(B)** VGG-16(Depth images). **(C)** VGG-16(NIR images). **(D)** ResNet-50 (RGB images). **(E)** ResNet-50 (Depth images). **(F)** ResNet-50 (NIR images). Classes 1, 2, 3, 4 and 5 represent severely irrigated deficit, slightly irrigated deficit, moderately irrigated, slightly over-irrigated, and severely over-irrigated of tomato water status, respectively.

The VGG-16 and ResNet-50 networks were trained using RGB images, depth images and NIR images, respectively. Hyperparameter settings: initial learning rate were set to 0.001, and mini-batch size was set to 32. Cross-entropy loss function was used to represent the loss function and Adam optimizer was adopted as the optimizer. To ensure the effect of feature extraction and speed up the training of the network, the weights of the main part of the feature extraction network of the network model were first frozen and trained using the official model pre-training weights. After 50 iterations, they were unfrozen and the training was ended with 30 more iterations. The accuracy of RGB images and NIR images on VGG-16 and ResNet-50 on the corresponding test sets are shown in **Table 1**. **Figure 8** shows the detection results of the tomato water status detection model constructed using one kind of image.

**Table 1:** Accuracy of detection model of tomato water status based on single-modal deep learning.

Model name	RGB images		Depth images		NIR images	
	Correct number of classifications	Accuracy	Correct number of classifications	Accuracy	Correct number of classifications	Accuracy
VGG-16	5876	90.68%	5765	88.97%	5904	91.11%
ResNet-50	5967	92.08%	5868	90.56%	6032	93.09%

The total number of samples in the test set is 6480.

It can be seen from **Table 1** that among the two models, the training using ResNet-50 had a higher accuracy, the major reason was that the ResNet-50 network had more layers compared to the VGG-16 network and used residual blocks without gradient disappearance or gradient explosion. In the same deep learning network, the NIR images had the highest accuracy in detecting the water status of tomatoes and the depth images had the lowest. The NIR images were more sensitive to water changes in the crops (Peng et al., 2005); the RGB images were mainly based on tomato plant texture and color, so the leaf texture and color would only change significantly when the crop experienced severe water shortage. In comparison with the NIR images and RGB images, the depth images contained more complex information but the CNNs was slightly less effective in extracting features from the depth images, so the accuracy of the model was lower compared to the results obtained by using RGB images and NIR images. The RGB images of tomato leaves were first segmented by using Mask R-CNN for instance segmentation, and then separately classified using VGG-16 with an accuracy of 89.09%, which was slightly lower than that of this paper(2020). It might be illustrated by that the overfitting occurred in Qihui Zhao’s study (Zhao et al., 2012), while the amount of data in this paper was relatively large and no overfitting occurred.

### 3.3 Multimodal deep learning network

The initial learning rate of the tomato water status detection network was set to 0.001 and the mini-batch size was set to 32. To ensure the effectiveness of the model in extracting features and speed up the training, the training was first conducted using the official pre-trained weights, and the weight parameters of its backbone feature extraction part were frozen, and after 50 iterations, the weight parameters of the backbone feature extraction part were unfrozen to continue the training, and the training was stopped after 30 iterations. When the training was completed, the accuracy of each model was obtained by experimenting with the test set.

**Figure 9:**
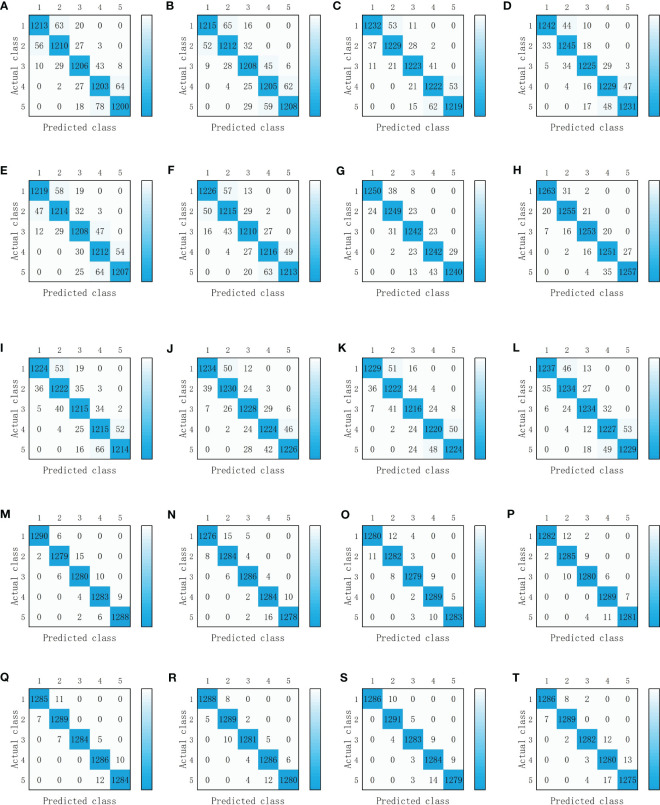
Detection results of multimodal deep learning models. **(A)** VGG16(RGB images) and VGG16(Depth images). **(B)** VGG16(RGB images) and ResNet50(Depth images). **(C)** VGG16(RGB images) and VGG16((NIR images). **(D)** VGG16(RGB images) and ResNet50((NIR images). **(E)** ResNet50(RGB images) and VGG16(Depth images). **(F)** ResNet50(RGB images) and ResNet50(Depth images). **(G)** ResNet50(RGB images) and VGG16((NIR images). **(H)** ResNet50(RGB images) and ResNet50((NIR images). **(I)** VGG16(Depth images) and VGG16((NIR images). **(J)** VGG16(Depth images) and ResNet50((NIR images). **(K)** ResNet50(Depth images) and VGG16((NIR images). **(L)** ResNet50(Depth images) and ResNet50((NIR images). **(M)** VGG16(RGB images), VGG16((NIR images) and VGG16((NIR images). **(N)** VGG16(RGB images), ResNet50((NIR images) and ResNet50((NIR images). **(O)** VGG16(RGB images), VGG16((NIR images) and VGG16((NIR images). **(P)** VGG16(RGB images), ResNet50((NIR images) and ResNet50((NIR images). **(Q)** ResNet50(RGB images), VGG16((NIR images) and VGG16((NIR images). **(R)** ResNet50(RGB images), ResNet50((NIR images) and ResNet50((NIR images). **(S)** ResNet50(RGB images), VGG16((NIR images) and VGG16((NIR images). **(T)** ResNet50(RGB images), ResNet50((NIR images) and ResNet50((NIR images). Classes 1, 2, 3, 4 and 5 represent severely irrigated deficit, slightly irrigated deficit, moderately irrigated, slightly over-irrigated, and severely over-irrigated of tomato water status, respectively.

The combined 20 tomato water status detection networks were trained, and the trained weights were tested on the test set after the training was completed, the accuracy of each detection network is shown in **Table 2** and the detection results are shown in **Figure 9**. Among the deep learning models built using two types of images, the highest accuracy was achieved by the combination of RGB images and NIR images extracted by ResNet-50, and the highest accuracy was achieved by the detection network built using three types of images extracted by ResNet-50 for RGB images and VGG-16 for depth and NIR images.

**Table 2:** Accuracy of detection model of tomato water status based on multimodal deep learning.

RGB images	Depth images	NIR images	Correct number of classifications	Accuracy
VGG-16	VGG-16	–	6032	93.09%
VGG-16	ResNet-50	–	6048	93.33%
VGG-16	–	VGG-16	6125	94.52%
VGG-16	–	ResNet-50	6172	95.25%
ResNet-50	VGG-16	–	6060	93.52%
ResNet-50	ResNet-50	–	6080	93.83%
ResNet-50	–	VGG-16	6223	96.03%
ResNet-50	–	ResNet-50	6279	96.90%
–	VGG-16	VGG-16	6090	93.98%
–	VGG-16	ResNet-50	6142	94.78%
–	ResNet-50	VGG-16	6111	94.31%
–	ResNet-50	ResNet-50	6161	95.08%
VGG-16	VGG-16	VGG-16	6420	99.07%
VGG-16	VGG-16	ResNet-50	6408	98.89%
VGG-16	ResNet-50	VGG-16	6413	98.97%
VGG-16	ResNet-50	ResNet-50	6417	99.03%
ResNet-50	VGG-16	VGG-16	6428	99.18%
ResNet-50	VGG-16	ResNet-50	6424	99.14%
ResNet-50	ResNet-50	VGG-16	6423	99.12%
ResNet-50	ResNet-50	ResNet-50	6412	98.95%

The total number of samples in the test set was 6480, and the “- “in the table indicates that the image was not used.

Results given in **Tables 1** and **2** disclosed that the tomato water status detection model using three kinds of images constituted the highest accuracy. The depth of each model using the three images had a large difference, but the difference in accuracy was not very large. This phenomenon may be resulted from that a high accuracy could be achieved by using a shallow depth VGG-16 to classify the features extracted from the three images after fusion, and even if a deeper depth ResNet-50 network was used, the accuracy would not be further improved.

## 4 Discussions

In this work, three kinds of image features were fused for deep learning, and the accuracy of the tomato water detection models built by the multimodal deep learning network was significantly improved compared to the single-modal deep learning network.

The accuracy of the deep learning model built with two images was about 5% higher than that of the single-modal model, and the accuracy of the deep learning model built with three images was about 5% higher than that of the deep learning model built with two kinds of images. A single RGB image, NIR image or depth image has its own limitations in characterizing plant water status information. For example, RGB images are mainly applied to extract color and texture information, but are easily affected by light and background; NIR images are sensitive to moisture changes but are susceptible to the influence of the environment; and the depth images are used to extract morphological information but are more complex. The use of multiple images can reflect the water status of the plant at more levels, so the accuracy of water status detection can be improved.

The accuracy of training with ResNet-50 was higher than that of training with VGG-16 under the same combination of images. The confusion matrix shown in **Figures 8**, **9** indicated that the single-modal water detection network produced the most errors in classifying two categories of severely irrigated deficit and slightly irrigated deficit and two categories of slightly over-irrigated and severely over-irrigated, which mainly attributed to the insignificant differences in crop color and morphology, so the detection accuracy of RGB images and depth images was lower. The fusion of three image features obtained by Gené-Mola et al. (2019) adapted a Faster R-CNN including five channels of images of color, depth and signal intensity for the recognition of apples and improved the composite metric over the Faster R-CNN containing only color, which also supported the above mentioned views.

## 5 Conclusions

This study introduced and compared single- modal and multimodal deep learning network to detect the water status of tomatoes. by fusing RGB, NIR and depth images. The experimental results showed that the accuracy of tomato water status detection based on single-mode deep learning ranged from 88.97% to 93.09%, while the accuracy of tomato water status detection based on multimode deep learning ranged from 93.09% to 99.18%. The multimodal deep learning significantly outperformed the single-modal deep learning. The optimal multimodal deep learning network combination for tomato water status detection was determined to use ResNet-50 to extract features from RGB images and VGG-16 to extract features from depth images and NIR images.

## Data availability statement

The original contributions presented in the study are included in the article/supplementary material. Further inquiries can be directed to the corresponding authors.

## Author contributions

ZZ, JM, WL, and HM conceived and designed the experiments. JM, WL, XZ, LH, and JN performed data collection and processing. ZZ, JM, and WL analyzed the data. ZZ, JM, WL, and QB drafted the manuscript. ZZ, JM, and HM revised the manuscript. All authors contributed to the article and approved the submitted version.
